# Maternal Overt Hypothyroidism and Pregnancy Complications: Insights from a Nationwide Cross-Sectional Study

**DOI:** 10.3390/jcm14155278

**Published:** 2025-07-25

**Authors:** Tamar Eshkoli, Nitzan Burrack, Adi Gordon-Irshai, Bracha Cohen, Merav Fraenkel, Uri Yoel

**Affiliations:** 1 Endocrinology Unit, Soroka University Medical Center, Beer-Sheva 84101, Israel; meravfra@gmail.com (M.F.); uriy@bgu.ac.il (U.Y.); 2 Department of Obstetrics and Gynecology, Soroka University Medical Center, Beer-Sheva 84101, Israel; 3 Faculty of Health Science, Ben-Gurion University of the Negev, Beer-Sheva 84101, Israel; burrack@post.bgu.ac.il (N.B.); adigordn@gmail.com (A.G.-I.); 4 Clinical Research Center, Soroka University Medical Center, Beer-Sheva 84101, Israel; brahaco@clalit.org.il

**Keywords:** overt hypothyroidism, pregnancy complications, TSH levels

## Abstract

**Background/Objectives**: Overt hypothyroidism during pregnancy has been linked to adverse outcomes, including preterm birth, low birth weight, and impaired fetal neurocognitive development. This study aimed to evaluate pregnancy complications in women with overt hypothyroidism (TSH ≥ 10) through a cross-sectional study. **Methods**: Data from 259,897 live-birth pregnancies (2013–2022) from Clalit Health Services (CHS) were analyzed. The study included all CHS-insured women aged ≥ 18 years with available TSH results during pregnancy. Overt hypothyroidism was defined as a mean TSH ≥ 10 mIU/L, while the euthyroid reference group had TSH levels < 4 mIU/L and no history of hypothyroidism or levothyroxine use. Cases of overt hypothyroidism were matched with 15 controls using propensity score-based matching. Covariates included maternal age, ethnicity, socioeconomic status, IVF use, recurrent pregnancy loss, and smoking. Pregnancy complications were compared between groups using descriptive statistics and univariate analysis. A quasi-Poisson regression model was used to assess complication risk in overt hypothyroidism versus matched controls. **Results**: The final analysis included 9125 euthyroid and 611 overt hypothyroid pregnancies, with comparable baseline characteristics between groups. No significant differences were found in maternal age, ethnicity, socioeconomic scores, IVF rates, recurrent pregnancy loss, diabetes, smoking, gestational age at delivery, or rates of preterm birth, pre-eclampsia, gestational diabetes, cesarean section, and intrauterine growth restriction. Overall, overt hypothyroidism was not associated with increased complications. Sensitivity analyses using maximum TSH levels during pregnancy showed a slightly elevated risk for pregnancy complications (IRR 1.1, CI 1.04–1.18; *p* = 0.002). **Conclusions**: Overt hypothyroidism was not associated with an increased risk of adverse pregnancy outcomes when adjusted for confounding factors, suggesting that treatment decisions should be made on an individual basis.

## 1. Introduction

Hypothyroidism is the most common pregnancy-related thyroid disorder, affecting 3–5% of all pregnant women [1]. Between 16 and 20 weeks of gestation, the fetus starts producing appreciable amounts of thyroid hormones required for optimal growth and the development of multiple organ systems, especially the brain. Prior to that, it depends largely on an adequate transplacental supply of maternal thyroid hormones from the early first trimester [2,3]. Maternal thyroid hormone production increases by approximately 50% during pregnancy due to hormonal and metabolic changes, including the effects of human chorionic gonadotropin (hCG) and rising estrogen levels [3].

Population-based studies demonstrate substantial differences in the TSH upper-reference limit during pregnancy. These differences may be partly attributable to differences in the iodine status between populations, as well as the TSH assays used for analysis [2]. There also seems to be important influences of body mass index, geography, and ethnicity upon TSH concentrations in pregnant women [2]. If available, trimester-specific reference ranges for serum thyroid-stimulating hormone (TSH) should be used for the interpretation of thyroid function tests during pregnancy [1].

Overt hypothyroidism is defined when an elevated TSH concentration is present at the same time as a low serum free thyroxine (FT4) concentration [4], or when serum TSH is above 10 mIU/L even if serum FT4 is normal [1]. Overt maternal hypothyroidism is associated with an increased risk of adverse pregnancy complications, such as increased risks of premature birth, low birth weight, pregnancy loss, and lower offspring intelligence quotient (IQ) [2]. Abalovich et al. [5] demonstrated that women with overt hypothyroidism carry an estimated 60% risk of fetal loss when not adequately controlled. Allan [6] similarly described an increased risk of fetal death among pregnant women with overt disease. Leung [7] demonstrated an increased risk of gestational hypertension in pregnant women with overt maternal hypothyroidism. When discussing the risk of preterm birth, it is essential to distinguish between cases where medical intervention was required to end the pregnancy (iatrogenic preterm births) and those that occurred spontaneously. The lack of this distinction in previous studies [8,9,10,11,12] investigating the association between first-trimester hypothyroidism and preterm birth limits the ability to draw clear conclusions about the risk. Additionally, these studies did not consider potential confounding factors or mediators, such as pre-eclampsia (PE) or the preterm rupture of membranes (PPROM). Similarly, in the case of low birth weight, retrospective studies did not account for conditions associated with placental insufficiency, such as hypertensive disorders, nor did they consider gestational age at delivery when calculating odds ratios. These limitations highlight the challenges in determining, from existing data, the primary etiologies of preterm delivery or low birth weight in cases of overt hypothyroidism [13].

Subclinical hypothyroidism (SCH) is more common than overt hypothyroidism and is typically defined as serum TSH above pregnancy-specific reference ranges, with normal free hormone levels [1]. While overt hypothyroidism is linked to adverse outcomes, the impact of SCH remains unclear. Some studies associate SCH with complications such as pregnancy loss, preterm delivery, gestational diabetes, pre-eclampsia, and neonatal complications, while others report no significant effects [14,15,16,17].

Therefore, our objective was to assess the relationship between TSH levels and pregnancy outcomes in women with overt hypothyroidism while considering established risk factors for pregnancy complications.

## 2. Materials and Methods

We conducted a retrospective cross-sectional study utilizing electronic medical records data from Clalit Health Services (CHS) members who underwent delivery at one of the 11 CHS network hospitals. CHS, Israel’s largest healthcare maintenance organization, covers more than 4.8 million insured individuals and maintains a notably stable, low annual turnover. Since 2000, CHS has operated an integrated electronic health records (EHR) system, encompassing patient information across primary, ambulatory, and in-hospital care settings. The EHR system is continually updated in real time through inputs from administrative, medical, and pharmaceutical systems. This distinctive database environment is subjected to ongoing monitoring and validation, ensuring both exposure and follow-up data’s reliability, consistency, and precision. The CHS population is essentially representative of the general Israeli population. We collected data from December 2012 until the end of 2022. The Soroka University Medical Center Institutional Review Board (IRB) approved the study in advance (Approval No. SOR 0143-21) and granted a waiver of informed consent in accordance with institutional guidelines. The study was conducted in compliance with the Declaration of Helsinki as revised in 2013. All patient records were anonymized and de-identified prior to analysis. Additionally, appropriate ethical approval was obtained, and all procedures followed the required ethical standards.

### 2.1. Study Population

The study included all women members of CHS aged ≥ 18 years with available TSH results taken during pregnancy and who remained members of CHS throughout the gestational period. Data on newborns’ weight and sex were coupled when available in case the newborn was a member of CHS. Women were classified as overt hypothyroid if they had a mean TSH during pregnancy of ≥10. The euthyroid reference group comprises women meeting all the following criteria: (1) a maximal TSH during pregnancy < 4, (2) no prior diagnosis of hypothyroidism (ICD-9 code) in the 21 months prior to delivery, and (3) no prior purchase of levothyroxine. To assess the association of transient hypothyroidism during pregnancy with obstetric complications, we analyzed a cohort of women separately according to their max TSH value during pregnancy > 10, matched to healthy controls meeting the above three criteria.

### 2.2. Data Sources and Definitions

We evaluated integrated patient-level data maintained by CHS from the primary care, ambulatory, and hospital care databases. The former contains demographic, medical history, and laboratory tests, while the latter includes hospitalization data and outpatient services. We extracted our data utilizing the Clalit Research Data sharing platform powered by MDClone. We extracted the following sociodemographic data: age, ethnicity, and socioeconomic status score (ranging from 1 [lowest] to 5 [highest]), assigned by the Israeli Central Bureau of Statistics, according to the patient’s home address. Additionally, we collected information regarding smoking status.

We evaluated the exposure to levothyroxine (ATC: H03AA01) during pregnancy by the linked dispenses registry, which includes drugs purchased from both private and CHS pharmacies. First, the total quantity of drugs dispensed was provided by the daily defined dose (DDD) of levothyroxine during pregnancy. Second, we divided this number by 0.66 to present the number of tabs in the commonly purchased form of 100 mcg. Lastly, we divide this number by the number of days of gestation (Gestation Week × 7), resulting in the daily average number of 100 mcg pills purchased by the women/patient.

### 2.3. Study Outcomes

Complications during pregnancy and delivery were assessed according to mean TSH exposure during gestation. Pre- and perinatal complications included placenta accreta, placenta previa, pre-eclampsia, severe pre-eclampsia, gestational hypertension, gestational diabetic mellitus (GDM), preterm premature rupture of membranes (PPROM), cesarean delivery, labor induction by medication, intrauterine growth restriction (IUGR), and polyhydramnios. Preterm delivery was defined by birth at <37 weeks.

### 2.4. Statistical Analysis

Following the application of inclusion and exclusion criteria, the study population was stratified into two cohorts: the exposed group, comprising women with mean TSH measurements ≥ 10 during gestation, and the control group, consisting of euthyroid women. To investigate potential pregnancy complications among women with overt hypothyroidism compared to euthyroid controls, propensity score-based matching was employed. Each woman diagnosed with overt hypothyroidism was matched with 15 euthyroid controls. Matching was based on key variables including maternal age, ethnicity, socioeconomic status, in vitro fertilization (IVF) utilization in the current pregnancy, history of recurrent pregnancy loss, and smoking status.

Univariate analysis results are presented as means ± SD for normally distributed variables. Categorical variables are reported as counts and proportions out of available cases. Differences in quantitative variables between groups were analyzed using an unpaired T-test or the nonparametric Kruskal–Wallis test. We assessed the risk of complications for overt hypothyroidism versus our matched controls by a multivariable quasi-Poisson regression modeling with the count of 14 different complications as the dependent variables: IUGR, preterm birth (<37 weeks), pre-eclampsia, severe pre-eclampsia, eclampsia, PPROM, GDM, gestational hypertension, postpartum hemorrhage (PPH), oligohydramnios, polyhydramnios, placenta previa, placenta accreta, and low birth weight (<2500 g). We adjusted the results to maternal age and other variables if were significantly different between the groups. We reported the result as the incidence rate ratio (IRR) with 95% confidence interval. For all analyses, significance was set at a two-sided *p*-value of <0.05. Statistical analyses were performed using R: Core Team, statistical software version 4.1.2 (R Foundation for Statistical Computing, Vienna, Austria).

## 3. Results

### 3.1. Description of the Study Population

#### 3.1.1. Cohort Description

**Figure 1:**
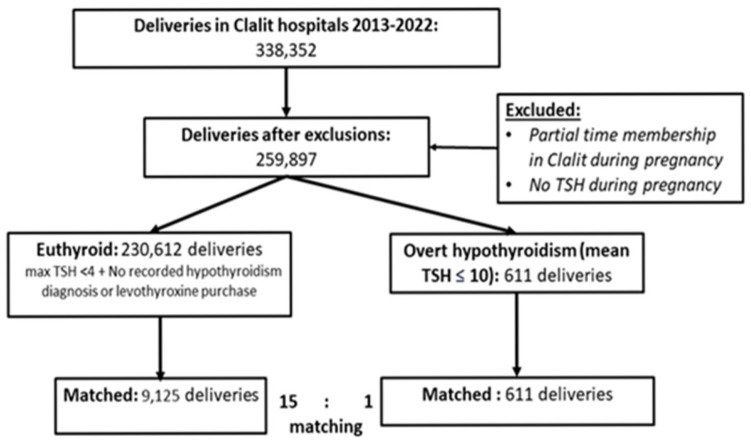
Flow chart of the study population.

A total of 259,897 deliveries with available thyroid function data during pregnancy were identified in Clalit hospitals between 2013 and 2022 (Figure 1). Among them, 230,612 were classified as euthyroid and 611 as overt hypothyroid based on predefined criteria.

#### 3.1.2. Matching Process

Matching was performed in a 15:1 ratio, resulting in a final cohort of 9125 deliveries of euthyroid women and 611 deliveries of women with overt hypothyroidism during pregnancy.

### 3.2. Baseline Characteristics

#### 3.2.1. Demographic and Clinical Characteristics

The baseline characteristics of the two groups were comparable, as expected due to the matching process. The mean maternal age was approximately 31 years. The majority of participants were of Arab ethnicity (about 55%).

#### 3.2.2. Lifestyle and Socioeconomic Variables

Socioeconomic status was evenly distributed, with nearly 50% in the Low or Very Low categories. Most women were non-smokers (83%); 7% had a history of smoking, and 5.8% were current smokers. Rates of in vitro fertilization, recurrent pregnancy loss, and diabetes mellitus did not differ significantly between the groups (Table 1).

### 3.3. Thyroid Function Tests

#### 3.3.1. TSH Levels Across Pregnancy

As expected, mean TSH levels were significantly higher in the overt hypothyroid group compared to controls (25.76 ± 30.99 vs. 1.59 ± 0.80). Maximal TSH values remained elevated throughout pregnancy in the hypothyroid group. Among these women, TSH levels decreased across trimesters (mean: first > second > third; approximately 50 > 24 > 19), suggesting effective treatment.

#### 3.3.2. Levothyroxine Treatment

The mean daily defined dose (DDD) of levothyroxine was 83.11 ± 66.06 mcg. Women with overt hypothyroidism underwent more frequent TSH testing (4.02 ± 2.49 vs. 1.42 ± 0.75; *p* < 0.001).

#### 3.3.3. Thyroid Antibodies and Test Frequency

Thyroid antibody levels were higher in the overt hypothyroidism group, as expected (Table 2).

### 3.4. Pregnancy Outcomes

#### 3.4.1. Overall Pregnancy Complications

Pregnancy characteristics and outcomes are presented in Table 3. Gestational age at delivery was similar between the groups. There were no significant differences in the rates of complications such as preterm delivery, pre-eclampsia, gestational diabetes, cesarean section, or intrauterine growth restriction.

#### 3.4.2. Statistical Analysis of Complication Rates

We compared the incidence of 14 different pregnancy complications between groups. Using a quasi-Poisson regression model adjusted for maternal age, there was no significant increase in complications among the overt hypothyroid group (IRR 1.02; 95% CI: 0.89–1.16) (Table 4).

### 3.5. Sensitivity Analyses

#### 3.5.1. Alternative Definition of Overt Hypothyroidism

We replicated our analysis on a parallel cohort where overt hypothyroidism was defined by the maximal TSH level during pregnancy (TSH > 10 mIU/L at any time during pregnancy). This yielded 2051 women, who were then matched with euthyroid controls (Table 5, Table 6 and Table 7).

#### 3.5.2. Specific Adverse Outcomes in Sensitivity Analysis

In this analysis, we observed a slightly elevated risk for pregnancy complications (IRR 1.1; Table 8). Specific adverse outcomes, including severe pre-eclampsia, preterm labor, oligohydramnios, polyhydramnios, cesarean delivery, and low birth weight, were more frequent among women with overt hypothyroidism (Table 8).

## 4. Discussion

Our study aimed to assess the association of overt hypothyroidism with pregnancy complications, while accounting for established risk factors. The findings of this study did not demonstrate a significant increase in the risk of adverse pregnancy outcomes among women with overt hypothyroidism compared to matched controls when adjusted for confounding factors. Specifically, no significant differences were observed in gestational age at delivery, rates of preterm birth, pre-eclampsia, gestational diabetes mellitus, cesarean section, or intrauterine growth restriction. Although the sensitivity analysis using maximal TSH levels suggested a slightly increased risk, the primary analysis based on mean TSH did not. Our findings emphasize that the mean TSH level throughout pregnancy provides a more stable and representative measure of thyroid function than a single maximal TSH value. The slight increase in risk observed in the sensitivity analysis using maximal TSH highlights the variability of thyroid status during pregnancy and the need for the cautious interpretation of isolated elevated TSH measurements.

Our results are consistent with Hirsch D et al. [18], who investigated 103 pregnancies in women with TSH levels exceeding 20.0 mIU/L. Despite high TSH values (median of 32.95 mIU/L) and all patients receiving levothyroxine therapy, adverse pregnancy outcomes—including abortions (7.8%) and preterm deliveries (2.9%)—were not significantly different from those in the euthyroid group.

Regarding miscarriage, our data could not capture early losses. However, previous studies have shown mixed results. Montoro et al. [19] described successful pregnancies despite severe hypothyroidism, while Abalovich et al. [5] reported increased miscarriage rates in inadequately treated cases. These findings suggest that proper management may mitigate risks, aligning with our observation that among those progressing beyond early pregnancy, no elevated complication risk was found.

In contrast, other studies reported increased risks. Leung et al. [7] observed a 22% incidence of gestational hypertension in overt hypothyroid patients, higher than in euthyroid or subclinical hypothyroid women. This discrepancy may stem from the small sample size (n = 23 overt cases) and differences in study design.

Similarly, Allan et al. [6] found an association between TSH > 10.0 mIU/L and fetal death in 37 women. However, this study relied on a single second-trimester TSH measurement, without follow-up or levothyroxine data, limiting its comparability to our longitudinal analysis.

Casey et al. [15] reported an increased risk of placental abruption and preterm birth among women with subclinical hypothyroidism but did not provide outcomes specifically for the subgroup with TSH > 10.0 mIU/L (n = 50). Thus, direct comparison to overt hypothyroid cases is limited.

A large retrospective cohort by Knøsgaard et al. [16] involving nearly 15,000 pregnancies found that women with TSH > 10 mIU/L in early pregnancy had higher rates of spontaneous abortion and preterm birth. Our findings differ, possibly due to differences in population, treatment rates, and study design.

Recent meta-analyses have focused primarily on subclinical hypothyroidism and thyroid autoimmunity [17,20,21,22].

Based on a systematic review [17], pregnant women with subclinical hypothyroidism or thyroid antibodies face elevated risks of complications, notably including pre-eclampsia, perinatal mortality, and recurrent miscarriage. Most of the studies included in this meta-analysis involved cases where the TSH was below 6. Based on the findings of those studies, it was extrapolated that these complications are also present in cases of overt hypothyroidism [20]. Moreover, in another meta-analysis, it was demonstrated that among pregnant women without overt thyroid disease, subclinical hypothyroidism, isolated hypothyroxinemia, and TPO antibody positivity were significantly associated with a higher risk of preterm birth [21]. Another meta-analysis [22] that investigated the relationship between maternal thyroid function and gestational hypertension and pre-eclampsia did not include cases of overt hypothyroidism.

Despite the lack of randomized controlled trials, clinical guidelines advocate for early and strict levothyroxine treatment in overt hypothyroidism during pregnancy to maintain TSH levels below 2.5 in the first trimester and below 3 thereafter [23]. Our findings support this recommendation, showing no increased complications in the majority of treated women. In our cohort, over 80% of women with TSH > 10 received levothyroxine (Table 2 and Table 6). Even if complete normalization was not achieved, partial treatment likely conferred sufficient protection.

Notably, approximately 15–20% of women with overt hypothyroidism in our cohort did not receive levothyroxine treatment during pregnancy. This could be due to delayed diagnosis, patient non-adherence, or incomplete data capture in the electronic health records. These factors represent real-world clinical challenges and underline the need for improved screening and management protocols.

The strengths of our study include its large sample size and extensive data coverage through an integrated electronic health records system. The study population’s representation of the general Israeli population adds external validity to the findings. The comprehensive approach in considering multiple risk factors and conducting sensitivity analyses adds depth to the investigation. Another strong aspect is the comparison group of women with hypothyroidism, relying on their thyroid function during pregnancy, and detected by a propensity score-based matching method, based on a large national cohort.

Limitations of the study include its retrospective design, reliance on electronic medical records, potential selection biases of women who performed TSH exams during pregnancy, and inability to distinguish spontaneous from iatrogenic preterm births. As miscarriage data were unavailable, this outcome could not be evaluated—though previous studies (e.g., Hirsch et al. [18]) did not find a strong association either.

Our sensitivity analysis, based on maximal TSH, suggested a modest increase in complications, emphasizing the need for further exploration and research in this complex relationship. Nevertheless, the mean TSH level throughout pregnancy is a more reliable indicator of thyroid homeostasis than the maximal TSH level.

## 5. Conclusions

In conclusion, overt hypothyroidism—defined by a mean TSH ≥ 10 in pregnancies progressing beyond early gestation—may not significantly increase adverse pregnancy outcomes when appropriately managed. These findings provide reassurance to clinicians and patients and highlight the importance of ongoing monitoring. Future studies may benefit from a prospective design to strengthen the evidence on this topic.

## Figures and Tables

**Table 1:** Background characteristics by mean TSH levels.

Characteristic	Euthyroidism, N = 9125	Overt hypothyroidism, N = 611	*p*-Value
Maternal Age	30.96 ± 5.54	31.03 ± 5.53	0.73
Ethnicity			0.99
Jews	3930 (43.1%)	265 (43.4%)	
Arabs	5034 (55.2%)	335 (54.8%)	
Unknown	161 (1.8%)	11 (1.8%)	
Socioeconomic Status			>0.99
Very High	368 (4.0%)	23 (3.8%)	
High	1031 (11.3%)	70 (11.5%)	
Medium	1409 (15.4%)	95 (15.5%)	
Low	2001 (21.9%)	132 (21.6%)	
Very Low	2131 (23.4%)	143 (23.4%)	
No Data	2185 (23.9%)	148 (24.2%)	
In Vitro Fertilization	204 (2.2%)	15 (2.5%)	0.72
Recurrent Pregnancy Loss	229 (2.5%)	17 (2.8%)	0.68
Diabetes Mellitus	191 (2.1%)	13 (2.1%)	0.95
Smoking			0.68
Unknown	315 (3.5%)	19 (3.1%)	
Past	634 (6.9%)	44 (7.2%)	
Current	527 (5.8%)	42 (6.9%)	
Never	7649 (83.8%)	506 (82.8%)	

**Table 2:** Laboratory characteristics and medication dispensation by mean TSH levels.

	Euthyroidism, N = 9125	Overt Hypothyroidism, N = 611	
Mean T4			<0.001
Mean ± SD	1.10 ± 0.18	1.00 ± 0.72	
Median (IQR)	1 (1.00, 1.20)	1 (0.85, 1.10)	
Mean TSH			<0.001
Mean ± SD	1.59 ± 0.80	25.59 ± 30.72	
Median (IQR)	2 (1.00, 2.09)	15 (11.70, 24.82)	
Count TSH			<0.001
Mean ± SD	1.42 ± 0.75	4.06 ± 2.53	
Median (IQR)	1 (1.00, 2.00)	4 (2.00, 6.00)	
Count T4			<0.001
Mean ± SD	0.38 ± 0.76	3.71 ± 2.42	
Median (IQR)	0 (0.00, 1.00)	3 (2.00, 5.00)	
Daily dose, Mean ± SD (N)	0.00 ± 0.00	83.42 ± 66.15	<0.001
Treated with levothyroxine (n, %)	0 (0.0%)	510 (83.5%)	<0.001
Max TSH 1T, Mean ± SD (N)	1.50 ± 0.86 (6138)	50.15 ± 57.56 (478)	<0.001
Max TSH 2T, Mean ± SD (N)	1.71 ± 0.80 (3932)	23.89 ± 38.90 (486)	<0.001
Max TSH 3T, Mean ± SD (N)	1.79 ± 0.80 (1676)	19.03 ± 40.14 (411)	<0.001
Anti-TPO, n (%)			<0.001
NEGATIVE	362 (4.0%)	19 (3.1%)	
Not tested	8255 (90.5%)	183 (30.0%)	
POSITIVE	508 (5.6%)	409 (66.9%)	
THYROGLOBULIN AB *, n (%)			<0.001
NEGATIVE	521 (5.7%)	101 (16.5%)	
POSITIVE	347 (3.8%)	321 (52.5%)	
Not tested	8257 (90.5%)	189 (30.9%)	

* AB: antibodies.

**Table 3:** Perinatal Outcomes by Mean TSH Levels.

Characteristic	0, N = 9125	4, N = 611	*p*-Value ^1^
Placenta accreta, n (%)	2 (0.0%)	0 (0.0%)	>0.99
Placenta previa, n (%)	114 (1.2%)	10 (1.6%)	0.41
Pre-eclampsia, n (%)	265 (2.9%)	14 (2.3%)	0.38
Severe pre-eclampsia, n (%)	81 (0.9%)	5 (0.8%)	0.86
Gestational hypertension, n (%)	113 (1.2%)	2 (0.3%)	0.044
Gestational diabetes mellitus, n (%)	733 (8.0%)	44 (7.2%)	0.46
Preterm premature rupture of membranes, n (%)	1287 (14.1%)	85 (13.9%)	0.89
Cesarean delivery, n (%)	1792 (19.6%)	126 (20.6%)	0.55
Preterm labor, n (%)	664 (7.3%)	55 (9.0%)	0.11
Labor induction, n (%)	1869 (20.5%)	112 (18.3%)	0.20
Assisted delivery, n (%)	369 (4.0%)	24 (3.9%)	0.89
Intrauterine growth restriction, n (%)	387 (4.2%)	26 (4.3%)	0.99
Polyhydramnios, n (%)	206 (2.3%)	20 (3.3%)	0.11
Oligohydramnios, n (%)	329 (3.6%)	16 (2.6%)	0.20
Postpartum hemorrhage, n (%)	224 (2.5%)	22 (3.6%)	0.081
OB/GYN visits count			0.24
Mean ± SD	9.38 ± 5.32	9.02 ± 5.08	
Median (IQR)	9.00 (6.00, 12)	9.00 (5.00, 12)	
Primary physician visits			<0.001
Mean ± SD	6.66 ± 5.75	9.28 ± 6.00	
Median (IQR)	5.00 (2.00, 9)	8.00 (5.00, 13)	
Hospital admissions during pregnancy			0.47
Mean ± SD	0.22 ± 0.78	0.23 ± 0.69	
Median (IQR)	0.00 (0.00, 0)	0.00 (0.00, 0)	
Number of live births, n (%)			0.48
0	35 (0.4%)	3 (0.5%)	
1	8873 (97.2%)	589 (96.4%)	
2	214 (2.3%)	19 (3.1%)	
3	3 (0.0%)	0 (0.0%)	
Birth week, mean ± SD	38.78 ± 1.92	38.80 ± 1.99	0.57
Low birth weight (< 2500 g), n/N (%)	674/8896 (7.6%)	46/597 (7.7%)	0.91

^1^ Fisher’s exact test; Pearson’s Chi-squared test; Wilcoxon rank-sum test.

**Table 4:** A quasi-Poisson regression model of pregnancy complications by mean TSH levels in women with overt hypothyroidism vs. euthyroid women.

Characteristic	IRR (95% CI) ^1^	*p*-Value
Control	—	
Mean TSH > 10	1.01 (0.89 to 1.15)	0.86
Age	1.02 (1.02 to 1.03)	<0.001

^1^ IRR = Incidence Rate Ratio, CI = Confidence Interval.

**Table 5:** Background characteristics by maximal TSH.

Characteristic	Euthyroidism, N = 30,739	Overt Hypothyroidism, N = 2051	*p*-Value
Maternal Age	31.75 ± 5.44	31.81 ± 5.49	0.76
Ethnicity			0.57
Jews	18,339 (59.7%)	1223 (59.6%)	
Arabs	11,575 (37.7%)	765 (37.3%)	
Unknown	825 (2.7%)	63 (3.1%)	
Socioeconomic Status			>0.99
Very High	2323 (7.6%)	154 (7.5%)	
High	7126 (23.2%)	472 (23.0%)	
Medium	6145 (20.0%)	411 (20.0%)	
Low	6771 (22.0%)	452 (22.0%)	
Very Low	4093 (13.3%)	273 (13.3%)	
No Data	4281 (13.9%)	289 (14.1%)	
In Vitro Fertilization	1503 (4.9%)	109 (5.3%)	0.39
Recurrent Pregnancy Loss	812 (2.6%)	61 (3.0%)	0.37
Diabetes Mellitus	576 (1.9%)	47 (2.3%)	0.18
Smoking			0.89
Unknown	1691 (5.5%)	114 (5.6%)	
Past	3235 (10.5%)	214 (10.4%)	
Current	1807 (5.9%)	129 (6.3%)	
Never	24,006 (78.1%)	1594 (77.7%)	

**Table 6:** Laboratory characteristics and medication dispensation by maximal TSH levels.

	Euthyroidism, N = 30,739	Overt Hypothyroidism, N = 2051	
Mean T4			<0.001
Mean ± SD	1.10 ± 0.17	1.07 ± 0.42	
Median (IQR)	1 (1.00, 1.20)	1 (0.95, 1.19)	
Mean TSH			<0.001
Mean ± SD	1.58 ± 0.79	11.77 ± 19.09	
Median (IQR)	1 (0.99, 2.07)	7 (4.97, 11.00)	
Count TSH			<0.001
Mean ± SD	1.47 ± 0.81	5.27 ± 2.64	
Median (IQR)	1 (1.00, 2.00)	5 (3.00, 7.00)	
Count T4			<0.001
Mean ± SD	0.42 ± 0.81	4.45 ± 2.50	
Median (IQR)	0 (0.00, 1.00)	4 (3.00, 6.00)	
Daily dose, Mean ± SD (N)	0.00 ± 0.00	94.20 ± 67.87	<0.001
Treated with levothyroxine (n, %)	0 (0.0%)	1179 (83.5%)	<0.001
Max TSH 1T, Mean ± SD (N)	1.52 ± 0.85 (22,716)	22.91 ± 34.00 (1845)	<0.001
Max TSH 2T, Mean ± SD (N)	1.68 ± 0.80 (11,981)	10.97 ± 22.08 (1824)	<0.001
Max TSH 3T, Mean ± SD (N)	1.75 ± 0.80 (5641)	7.69 ± 21.38 (1644)	<0.001
Anti-TPO, n (%)			<0.001
NEGATIVE	1494 (4.9%)	100 (4.9%)	
Not tested	27,333 (88.9%)	555 (27.1%)	
POSITIVE	1912 (6.2%)	1396 (68.1%)	
THYROGLOBULIN AB *, n (%)			<0.001
NEGATIVE	2094 (6.8%)	425 (20.7%)	
POSITIVE	1215 (4.0%)	1042 (50.8%)	
Not tested	27,430 (89.2%)	584 (28.5%)	

* AB—Antibodies.

**Table 7:** Perinatal outcomes by maximal TSH levels.

Characteristic	Euthyroidism, N = 30,739	Overt Hypothyroidism, N = 2051	*p*-Value
Placenta accreta, n (%)	6 (0.0%)	0 (0.0%)	>0.99
Placenta previa, n (%)	398 (1.3%)	29 (1.4%)	0.64
Pre-eclampsia, n (%)	872 (2.8%)	64 (3.1%)	0.46
Severe pre-eclampsia, n (%)	318 (1.0%)	35 (1.7%)	0.004
Gestational hypertension, n (%)	534 (1.7%)	38 (1.9%)	0.70
Gestational diabetes mellitus, n (%)	2849 (9.3%)	216 (10.5%)	0.057
Preterm premature rupture of membranes, n (%)	4504 (14.7%)	305 (14.9%)	0.79
Cesarean delivery, n (%)	6260 (20.4%)	462 (22.5%)	0.019
Preterm labor, n (%)	2213 (7.2%)	185 (9.0%)	0.002
Labor induction, n (%)	6132 (19.9%)	374 (18.2%)	0.060
Assisted delivery, n (%)	1496 (4.9%)	111 (5.4%)	0.27
Intrauterine growth restriction, n (%)	1496 (4.9%)	117 (5.7%)	0.089
Polyhydramnios, n (%)	715 (2.3%)	68 (3.3%)	0.004
Oligohydramnios, n (%)	1120 (3.6%)	50 (2.4%)	0.004
Postpartum hemorrhage, n (%)	940 (3.1%)	58 (2.8%)	0.56
OB/GYN visits count			0.001
Mean ± SD	10.57 ± 5.65	10.99 ± 5.95	
Median (IQR)	10.00 (7.00, 13)	10.00 (7.00, 14)	
Family visits count			<0.001
Mean ± SD	6.82 ± 5.71	10.45 ± 6.42	
Median (IQR)	5.00 (3.00, 9)	10.00 (6.00, 14)	
Hospital admissions count			0.086
Mean ± SD	0.22 ± 0.71	0.23 ± 0.70	
Median (IQR)	0.00 (0.00, 0)	0.00 (0.00, 0)	
Number of live births, n (%)			0.60
0	78 (0.3%)	4 (0.2%)	
1	29,883 (97.2%)	1989 (97.0%)	
2	759 (2.5%)	58 (2.8%)	
3	19 (0.1%)	0 (0.0%)	
Birth week, mean ± SD	38.76 ± 1.87	38.67 ± 1.99	0.15
Low birth weight (< 2500 g), n/N (%)	2256/29,963 (7.5%)	187/2000 (9.4%)	0.003

**Table 8:** A quasi-Poisson regression model of pregnancy complications by maximal TSH levels in women with overt hypothyroidism vs. euthyroid women.

Characteristic	IRR (95% CI) ^1^	*p*-Value
Control	—	
Max TSH > 10	1.11 (1.04 to 1.18)	0.002
Age	1.03 (1.02 to 1.03)	<0.001

^1^ IRR = Incidence Rate Ratio, CI = Confidence Interval.

## Data Availability

The original contributions presented in this study are included in the article. Further inquiries can be directed to the corresponding author.
